# Senior food insecurity in the USA: a systematic literature review

**DOI:** 10.1017/S1368980022002415

**Published:** 2022-11-04

**Authors:** Bertille Octavie Mavegam Tango Assoumou, Courtney Coughenour, Amruta Godbole, Ian McDonough

**Affiliations:** 1 University of Nevada, Las Vegas School of Public Health, Department of Environmental and Occupational Health, Las Vegas, USA; 2 University of Nevada, Las Vegas School of Public Health, Department of Epidemiology and Biostatistics, Las Vegas, USA; 3 University of Nevada, Las Vegas Lee Business School, Department of Economics, Las Vegas, USA

**Keywords:** Older adults, Food security, Hunger, Public health, Social determinants of health, Health equity

## Abstract

**Objective:**

Understanding the factors associated with senior food insecurity is key to understanding senior-specific needs to develop targeted interventions and ultimately lower the prevalence and the incidence of food insecurity. We aimed to systematically review published literature and summarise the associated factors of food insecurity in older adults in the USA.

**Design:**

We searched PubMed, Scopus, Web of science, EconLit and JSTOR databases for peer-reviewed articles published in English between January 2005 and September 2019 that assessed food security or its associated factors for US adults aged 60 years and older. After a two-step screening process, twenty articles were retained and included in the review.

**Setting:**

NA

**Participants:**

NA

**Results:**

The majority of studies were cross-sectional (70 %), consisted of data from one state (60 %), and had large sample sizes. Food-insecure individuals were more likely to be younger, less educated, Black or African American, female, a current smoker, low income, and self-report fair/poor health, have chronic conditions, and utilise government assistance programmes. Food insecurity was associated with medication non-adherence, poor mental health outcomes and limitations in physical functioning. Results were mixed for overweight/obesity status. There was no discernable pattern related to the consistency of findings by the assessed quality of the included studies.

**Conclusions:**

Food insecurity is a prevalent and pervasive issue for older adults. The numerous correlates identified suggest that interventions aimed at enhancing food and nutrition safety net and medication assistance programmes are warranted, and upstream, systemic-level interventions may be best suited to deal with the correlates of food insecurity.

Household-level food insecurity is a major public health concern in the USA. Household food insecurity is defined as the absence of sufficient, reliable access to food due to a lack of money and/or resources^(1)^. An increasingly higher number of Americans are food-insecure. In the USA in 2019, 13·7 million people, or 10·5 % of all households, lived in food-insecure households, with 4·1 % of those being very low food-secure. Nearly 7 % of households with an older adult aged 65 years or older were food-insecure, and 7·2 % of households with an older adult living alone were food-insecure^(2)^. The COVID-19 pandemic disrupted income, employment and overall stability to a magnitude not seen in recent history, and food insecurity has risen substantially as a result. Estimates from October 2020 projected that rates were 4·1 percentage points higher than they were in 2018 for adults and nearly 5 % points higher for children resulting in 50·4 million food-insecure individuals^(3)^. As for older adults in particular, a survey from July 2020 indicated that the Meals on Wheels ‘programs …(were) serving an average of 77 % more meals and 47 % more seniors than they were March 1, (2020)’^(4)^.

Food insecurity is a critical public health concern, as it is known to have detrimental short-term and long-term health consequences. Food insecurity is associated with poor physical and mental health outcomes, and food-insecure people face significant unmet needs for chronic disease prevention^(5)^. Food insecurity is also associated with several chronic diseases including diabetes, depression, high blood pressure, CHD and chronic kidney disease and is associated with substantially higher healthcare costs^(6)^.

One relatively understudied group in the published literature^(1,7–12)^ facing food insecurity are older adults or the senior population. This is problematic given the well-known and severe consequences associated with food insecurity among seniors. Seniors are especially vulnerable given the increased risk for acute and chronic health conditions. For example, food-insecure seniors are 91 % more likely to have asthma, 64 % more likely to be diabetic and 57 % more likely to have congestive heart failure^(13)^. Additionally, a large percentage of seniors live on a fixed income and are often forced to make spending trade-offs^(3)^. In other words, they are forced to choose between paying for food and paying for other necessities such as housing and/or transportation. The population of seniors is expected to grow as people continue to live longer. For example, the 85 and older population is expected to see a 123 % increase by 2040^(14)^. Of the current senior population in the USA, 7·3 %, or 5·3 million, were estimated to be food-insecure in 2018^(15)^.

A better understanding of the factors associated with senior food insecurity is key to understanding senior-specific needs to develop targeted interventions and ultimately lower the prevalence and the incidence of food insecurity. To our knowledge, no study has yet to systematically examine the published literature to identify associated factors of senior food insecurity in the USA. The purpose of this study is to systematically review the literature and summarise the factors associated with senior food insecurity in the USA.

## Methods

### Search Strategy

A search was conducted in five electronic databases to identify articles that examined food insecurity and its correlates among older adults in the USA. The databases included PubMed, Scopus, Web of science, EconLit and JSTOR. In order to conduct the search, the following MeSH terms were used: Senior OR old* adults OR elderly OR ageing adults OR aged AND ‘food insecurity’ OR ‘food security’ AND ‘United States’.

### Inclusion and exclusion criteria

Studies included in this review were those that assessed food security, and its correlates specifically among people aged 60 years and older were peer-reviewed and published in English, conducted in the USA, and published between January 2005 and September 2019. Other inclusion criteria included studies that assessed food insecurity as the dependent or independent variable of interest. Studies excluded from this review were those that were published before 2005, did not examine food security rates for people aged 60 years and older explicitly, or were conducted outside of the USA. Qualitative studies were also excluded from this review.

### Data screening and extraction

**Fig. 1:**
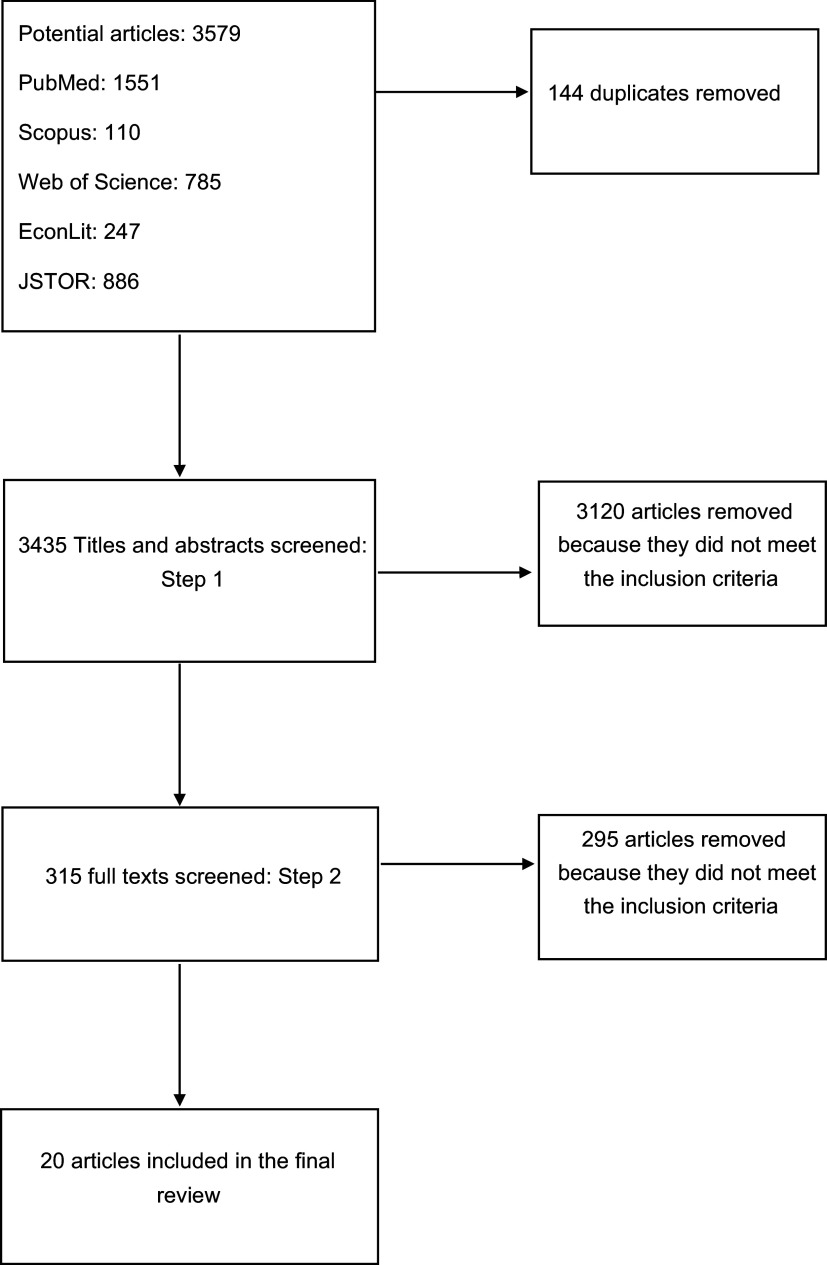
PRISMA diagram of articles in the systematic review of determinants of senior food insecurity. PRISMA, Preferred Reporting Items for Systematic Reviews and Meta-Analyses

All articles resulting from the five-database search were exported into the reference management software RefWorks, and duplicates were identified and removed. Data screening was conducted in two steps. In step 1, articles’ titles and abstracts were screened for eligibility. Titles and abstracts that met the inclusion criteria were moved to step 2. The full texts of eligible articles from step 1 were screened in step 2 to assess their adherence to the inclusion criteria. The screening process was conducted by three researchers to ensure quality and accuracy. First, two researchers independently reviewed titles and abstracts. Any discordances were then resolved by a third researcher. Second, two researchers independently examined the full texts of articles that were ‘screened in’ in step 1. Any discordances were again resolved by a third researcher. A Preferred Reporting Items for Systematic Reviews and Meta-Analyses (PRISMA) diagram summarising the results from the screening process is found in Fig. 1. Data of interest was extracted from all eligible articles by one researcher and reviewed for accuracy by a second researcher. Data extracted included the study authors, location, design, population, results, type of dataset and reported limitations. See full results in Table 1.

**Table 1:** Data extraction from all twenty studies meeting the inclusion criteria

Study	Data source	Study design	Sample description	Sample demographics	FI measure	FI prevalence	Outcomes
Afulani, P., Herman, D., Coleman-Jensen, A., & Harrison, G. G. (2015). Food Insecurity and Health Outcomes Among Older Adults: The Role of Cost-Related Medication Underuse. *Journal of Nutrition in Gerontology and Geriatrics*, *34*(3), 319–342. https://doi.org/10.1080/21551197.2015.1054575	Combined sample of 2011 and 2012 National Health Interview Survey (NHIS), national, USA	Cross-sectional	Annual, nationally representative, cross-sectional survey that provides data on the health of the non-institutionalised, civilian population in the USA; subsample of adults with complete income data and age >= 65 years.	*n* 10 401, 65+ years, 10·7 % Hispanic, 70·1 % non-Hispanic White, 13·5 % Non-Hispanic Black, 5·7 % Non-Hispanic Asian/other, 58·2 % female and 41·8 % male.	USDA Household Food Security Survey Module (HFSSM) ten-item survey, categories of food security – high, marginal, low and very low.	7·1 %	- FI individuals more likely to skip medications, take less medicine, delay prescription filling, take lower cost medication and be unable to afford medications.
Bengle, R., Sinnett, S., Johnson, T., Johnson, M. A., Brown, A., & Lee, J. S. (2010). Food insecurity is associated with cost-related medication non-adherence in community-dwelling, low-income older adults in Georgia. *Journal of Nutrition for the Elderly*, *29*(2), 170–191. https://doi.org/10.1080/01639361003772400	Baseline wave of the Georgia Advanced Performance Outcomes Measures Project – 6, state, USA	Cross- sectional	Data collected from low-income, non-institutionalised Georgia Older Americans Act Nutrition Program (OAANP) participants and waitlisted individuals aged 60 years and older; subsample without sensory impairment, with knowledge of English, and presence of complete address information.	*n* 1000, 60+ years, mean age = 75 ± 9·08, 58·2 % White, 25·8 % African-American, 16 % Asian/American Indian/Alaska Native/Other, 68·4 % female and 31·6 % male.	USDA Household Food Security Survey Module (HFSSM) six-item survey, categories of food security – food-secure and food-insecure.	49·7 %	- FI individuals more likely to be younger, Black or African Americans, and less educated, have low income (< $20 000/year), be food stamp recipients, be congregate meal participants, have fair/poor self-reported health, have a history of heart attack, angina/CHD, stroke, or diabetes, and have 4–7 chronic diseases. - FI participants more likely to practice cost-related medication non-adherence.
Bhargava, V., & Lee, J. S. (2017). Food Insecurity and Health Care Utilization Among Older Adults. *Journal of Applied Gerontology*, *36*(12), 1415–1432. https://doi.org/10.1177/0733464815625835	Georgia Advanced Performance Outcomes Measures Project 6 –CMS (linked ‘Georgia aging services client data and Medicare claims data from the Centers for Medicare and Medicaid Services’), state, USA.	Cross-sectional and longitudinal	Data from Centers for Medicare and Medicaid Services (2008 data) linked to data from the Georgia Advanced Performance Outcomes Measures Project – 6; subsample of low-income, minority, non-institutionalised older adults 65+ years old and had complete information on key variables.	*n* 957, 65+ years, mean age = 76·9 years, 71·3 % White, 28·7 % Black/Other, 68·9 % female and 31·1 % male.	USDA Household Food Security Survey Module (HFSSM) six-item survey, categories of food security – food-secure and food-insecure.	48·3 %	- FI individuals more likely to have lower income (< $20 000/year), be younger, from racial minorities, less educated, have poor/fair self-reported health, have diabetes, be food stamp recipients and be on dual enrolled health insurance
Brewer, D. P., Catlett, C. S., Porter, K. N., Lee, J. S., Hausman, D. B., Reddy, S., & Johnson, M. A. (2010). Physical limitations contribute to food insecurity and the food insecurity-obesity paradox in older adults at senior centres in Georgia. *Journal of Nutrition for the Elderly*, *29*(2), 150–169. https://doi.org/10.1080/01639361003772343	40 senior centres (2007) in Georgia, state, USA.	Cross-sectional	Georgia adults from senior centres aged 60 years and older, participating in the Older Americans Act congregate meal-site programme; subsample had participants who were not homebound, had complete data from the survey, were either Black or White, and were not underweight.	*n* 621, 60+ years, 63·6 % White, 36·4 % Black, 82·9 % female and 17·1 % male.	USDA Household Food Security Survey Module (HFSSM) six-item survey, categories of food security – food-secure and food-insecure.	18·7 %	-FI individuals more likely to be younger, Black or African Americans, have higher BMI and waist circumference, have arthritis or joint pain, have poor/moderate physical function, and have weight-related disabilities. -FI individuals more likely to have BMI class 2 obesity and waist circumference class 1 and 2 obesity.
Brooks, J. M., Petersen, C. L., Titus, A. J., Umucu, E., Chiu, C., Bartels, S. J., & Batsis, J. A. (2019). Varying Levels of Food Insecurity Associated with Clinically Relevant Depressive Symptoms in U.S. Adults Aged 60 Years and Over: Results from the 2005–2014 National Health and Nutrition Survey. *Journal of Nutrition in Gerontology and Geriatrics*, *38*(3), 218–230. https://doi.org/10.1080/21551197.2019.1611520	National Health and Nutrition Examination Survey (NHANES) (2005–2014), national, USA.	Cross-sectional	Cross-sectional survey ‘representative of the civilian, non-institutionalised population of the United States’, subsample of 60+ adults without missing data.	*n* 7969, 60+ years, 53·4 % Non-Hispanic White, 20·9 % Non-Hispanic Black, 19·9 % Hispanic, 5·7 % others, 49·4 % males and 50·6 % females.	USDA Household Food Security Survey Module (HFSSM) ten-item survey, categories of food security – high, marginal, low and very low.	17·7 %	-Marginally food-secure participants more likely to be younger, female, Non-Hispanic White, married/living with a partner, non-smokers, not completed high school, have a BMI more than 30, and have hypertension, diabetes, arthritis, and more SNAP benefits than those with low or very low food security. - Very low, low and marginal food security associated with more clinically relevant depressive symptoms as compared with fully food-secure participants.
Brostow, D. P., Gunzburger, E., Abbate, L. M., Brenner, L. A., & Thomas, K. S. (2019). Mental Illness, Not Obesity Status, is Associated with Food Insecurity Among the Elderly in the Health and Retirement Study. *Journal of Nutrition in Gerontology and Geriatrics*, *38*(2), 149–172. https://doi.org/10.1080/21551197.2019.1565901	Health and Retirement study (HRS) 2012, national, USA.	Cross-sectional	Survey from ‘nationally representative longitudinal study of community dwelling adults aged 50 and older that is administered every two years via mail, telephone, or in-person interview’; subsample of participants linked to 2012 Core data, with complete information on food security, aged 65 years and older, complete BMI data, not living in assisted living or long-term care facilities.	*n* 2868, 65+ years, mean age = 75·7 years, 63 % female, 37 % male and 82·7 % White.	USDA Household Food Security Survey Module (HFSSM) six-item survey, categories of food security – high, marginal, low and very low.	17·9 %	- Current smoking status, non-White race, history of a heart condition, preventative health behaviours, and especially depression and self-report of a psychiatric diagnosis were all associated with increased odds of being food-insecure -’increasing weight was not associated with increased odds of food insecurity after adjustment for multiple variables.’
Duerr L. (2006). Prevalence of food insecurity and comprehensiveness of its measurement for older adult congregate meals programme participants. *Journal of Nutrition for the Elderly*, *25*(3–4), 121–146. https://doi.org/10.1300/j052v25n03_09	Area 7 Agency on Aging and Disabled’s 22 congregate meals programme sites in West Central Indiana, local, USA.	Exploratory	Subsample of community-dwelling older adults aged 60 years and older participating in congregate meals	*n* 189, 60+ years, 33 % males and 67 % females.	USDA Household Food Security Survey Module (HFSSM) eighteen-item survey, categories of food security-secure, insecure without hunger and insecure with hunger (moderate/severe).	19·6 %	- FI without hunger were more likely to be female, between 75–84 years old, and high school graduates. - FI with moderate hunger were more likely to be male, 60–74 years old and with less than high school education - FI with severe hunger were more likely to be male, less than high school educated and between 65 and 74 years old.
Frith, E., & Loprinzi, P. D. (2018). Food insecurity and cognitive function in older adults: Brief report. *Clinical Nutrition*, *37*(5), 1765–1768. https://doi.org/10.1016/j.clnu.2017.07.001	National Health and Nutrition Examination Survey (NHANES) (1999–2002), USA.	Cross-sectional	Cross-sectional survey ‘representative of the civilian, noninstitutionalised population of the United States’; subsample included non-institutionalised 60–85-year-old US civilians, with complete information on heart conditions.	*n* 1851, 60–85 years, mean age = 69·8 years, 83·6 % White, 16·4 % other races, 58·6 % female and 41·4 % male.	USDA Household Food Security Survey Module (HFSSM) eighteen-item survey, categories of food security – high, marginal, low and very low.	7 %	- ‘Individuals who were marginally food secure, foodinsecure without hunger, and foodinsecure with hunger had significantly lower cognitive function’.
Goldberg, S. L., & Mawn, B. E. (2015). Predictors of Food Insecurity among Older Adults in the USA. *Public Health Nursing*, *32*(5), 397–407. https://doi.org/10.1111/phn.12173	National Health and Nutrition Examination Survey (NHANES) (2007–2008), national, USA.	Cross-sectional	Cross-sectional survey ‘representative of the civilian, noninstitutionalised population of the United States’; subsample included adults 60 years of age and older.	*n* 2045, 60+ years, mean age = 70·2 years, 3·9 % Mexican-American, 3·2 % other Hispanic, 79·8 % White, 8·7 % Black, 4·4 % others/multi-racial, 55·9 % female and 44·1 % male.	USDA Household Food Security Survey Module (HFSSM) ten-item survey, categories of food security – food-secure and food-insecure.	9·1 %	- FI participants were more likely to be unmarried, Mexican-American, have a lower education level, require food stamps and have depression. - FI participants were less likely to have help with financial support and private insurance coverage.
Hernandez, D. C., Reesor, L., & Murillo, R. (2017). Gender Disparities in the Food Insecurity-Overweight and Food Insecurity-Obesity Paradox among Low-Income Older Adults. *Journal of the Academy of Nutrition and Dietetics*, *117*(7), 1087–1096. https://doi.org/10.1016/j.jand.2017.01.014	National Health Interview Survey (NHIS) data (2011–2012), national, USA.	Cross-sectional	‘NHIS uses a multistage probability sample survey design to acquire a representative sample of the US non-institutionalised civilian population’; subsample included adults aged 60 years and older, with normal to greater BMI, low income, and with all data present.	*n* 5506, 60+ years, mean age = 71·6 years, 65 % White, 14 % Bblack, 15 % Hispanic, 6 % others, 62 % females and 38 % males.	USDA Household Food Security Survey Module (HFSSM) ten-item survey, categories of food security – food-secure and food-insecure.	19 %	- ‘(FI) men had 42 % lower odds of being overweight compared with normal weight and 41 % lower odds of being overweight or obese compared with normal weight…’ - FI was not associated with weight in women
Holben, D. H. Barnett, M. A., & Holcomb, J.P. (2008). Food Insecurity Is Associated with Health Status of Older Adults Participating in the Commodity Supplemental Food Program in a Rural Appalachian Ohio County. *Journal of Hunger & Environmental Nutrition, 1:2,* 89–99. https://doi.org/10.1300/J477v01n02_06	Convenience sample from Commodity Supplemental Food Program (CSEP) from Athens County, Ohio, local, USA.	Cross-sectional	The Commodity Supplemental Food Program (CSFP) is a food assistance programme that works to improve the health of older adults, as well as the health of pregnant and breast-feeding women, other new mothers up to 1-year postpartum, infants, and children up to the age of 6 years by supplementing their diets with nutritious USDA commodity foods; subsample of older adults 60 years and older receiving Commodity Supplemental Food Program (CSFP) benefits and living in households located in Athens County, Ohio	*n* 91, 60+ years, mean age = 71 years, 61·5 % female and 38·5 % male.	USDA Household Food Security Survey Module (HFSSM) ten-item survey, categories of food security – food-secure, food-insecure without hunger and food-secure with hunger.	49·5 %	- Participants with household FI were more likely to have poorer health scores (lower physical functioning, greater body pain, poorer general health, social functioning and mental health) than participants from food-secure households.
Jackson, J. A., Branscum, A., Tang, A., & Smit, E. (2019). Food insecurity and physical functioning limitations among older US adults. *Preventive Medicine Reports*, *14*, 100 829. https://doi.org/10.1016/j.pmedr.2019.100829	National Health and Nutrition Examination Survey (NHANES) (2007–2012), national, USA	Cross-sectional	Cross-sectional survey ‘representative of the civilian, noninstitutionalized population of the United States’; subsample of 60+ adults without missing data.	*n* 5969, 60+ years, 78·7 % non-Hispanic White, 8·9 % non-Hispanic Black, 7·3 % Hispanic, 5·1 % other, 44·5 % male and 55·5 % female	USDA Household Food Security Survey Module (HFSSM) ten-item survey, categories of food security – high, marginal, low and very low.	12 %	-FI individuals were more likely to be younger, female, non-Hispanic White, and less educated, be obese and current smokers, and report three or more chronic diseases. - Physical functioning limitations increased as severity of food insecurity increased. - Participants with four or more physical limitations had greater odds of very low food security. - the odds of FI were greater for those with physical functioning limitations, > 3 chronic diseases and non-Whites, and lower for those > 70 years of age, females, Whites, and those with higher income
Johnson, C. M., Sharkey, J. R., & Dean, W. R. (2011). Indicators of material hardship and depressive symptoms among homebound older adults living in North Carolina. *Journal of Nutrition in Gerontology and Geriatrics*, *30*(2), 154–168. https://doi.org/10.1080/21551197.2011.566527	Nutrition and Function Study, North Carolina, state, USA.	Cross-sectional	NAFS was a university–community collaborative project between the School of Public Health at the University of North Carolina at Chapel Hill and the home-delivered meals component of the Older Americans Act Nutrition Programs in four North Carolina counties; subsample of homebound adults older than 60 years of age, residing in North Carolina, who were current home-delivered meals participants	*n* 345, 60+ years of age, mean age = 78·2 years, 48·7 % Black, 51·3 % White, 80·9 % female and 19·1 % male.	Four-question survey related to absence of food and resource allocation, categories of food security – food-secure, at risk of food insecurity, food-insecure.	40·8 %	- FI participants were five times more likely to report depressive symptoms than food-secure. - FI status was associated with greater number of depressive symptoms
Kihlström, L., Burris, M., Dobbins, J., McGrath, E., Renda, A., Cordier, T., Song, Y., Prendergast, K., Serrano Arce, K., Shannon, E., & Himmelgreen, D. (2019). Food Insecurity and Health-Related Quality of Life: A Cross-Sectional Analysis of Older Adults in Florida, U.S. *Ecology of Food and Nutrition*, *58*(1), 45–65. https://doi.org/10.1080/03670244.2018.1559160	Survey from the waiting rooms of three primary care clinics in West-Central Florida between May 2017 and October 2017, local, USA.	Cross-sectional	The three clinics (in contract with the study sponsor), which offered primary care services to adult patients, were selected to participate in this study based on their interest and ability to recruit patient participants. Sample included patients aged 65 years and older	*n* 234, 65+ years, mean age = 76·2 years, 60·7 % females, 39·3 % males, 10·3 % African-American, 41·5 % Hispanic/Latino, 43·2 % White/Caucasian, 5·1 % other.	USDA Household Food Security Survey Module (HFSSM) six-item survey, categories of food security – food-secure and food-insecure.	19·4 %	- FI individuals were more likely to be divorced/separated, have lower income, have more health conditions, not be on SNAP benefits, be lonely and have higher social support.-
Lee, J. S., Johnson, M. A., & Brown, A. (2011). Older Americans Act Nutrition Program improves participants’ food security in Georgia. *Journal of Nutrition in Gerontology and Geriatrics*, *30*(2), 122–139. https://doi.org/10.1080/21551197.2011.566526	Georgia Advanced Performance Outcomes Measures Project – 6, state, USA.	Longitudinal	The GA Advanced POMP 6 consisted of self-administered mail surveys completed by community-dwelling active and new OAANP participants and waitlisted persons; subsample had English literate and non-disables older adults.	*n* 717, 60+ years, mean age = 74·6 years, 70·9 % female, 29·1 % male, 65·3 % White and 33·2 % Black.	Modified version of the six-item USDA HFSSM, categories of food security – high, marginal, low and very low.	54 %	- Participants waitlisted for congregate meal and home-delivered meal plans were more likely to be FI than those already enrolled. - Those who were younger, Black, less educated, poor, receiving food stamps and self-reporting poorer health status were less likely to achieve food security than their counterparts.
Myles, T., Porter Starr, K. N., Johnson, K. B., Sun Lee, J., Fischer, J. G., & Ann Johnson, M. (2016). Food Insecurity and Eating Behavior Relationships Among Congregate Meal Participants in Georgia. *Journal of Nutrition in Gerontology and Geriatrics*, *35*(1), 32–42. https://doi.org/10.1080/21551197.2015.1125324	Four senior centres affiliated with the Northeast Georgia’s Area Agency on Aging, local, USA.	Cross-sectional	Congregate meal participants in northeast Georgia who were 60 years of age and older.	*n* 118, 60+ years, mean age = 75 years, 25 % male, 75 % female, 57 % White and 43 % Black.	Modified version of the six-item USDA HFSSM, categories of food security – high, marginal, low and very low.	59·3 %	- FI status was associated with cognitive restraint, and uncontrolled eating.
Redmond, M. L., Dong, F., Goetz, J., Jacobson, L. T., & Collins, T. C. (2016). Food Insecurity and Peripheral Arterial Disease in Older Adult Populations. *The Journal of Nutrition, Health & Aging*, *20*(10), 989–995. https://doi.org/10.1007/s12603-015-0639-0	National Health and Nutrition Examination Survey (NHANES) (1999–2004), national, USA.	Cross-sectional	Cross-sectional survey ‘representative of the civilian, noninstitutionalized population of the United States’; subsample of 60+ adults without missing data.	*n* 2027, 60+ years, 48·3 % male, 51·7 % female, 47·4 % non-Hispanic White, 17·9 % Non-Hispanic Black, 32·3 % Hispanic and 2·5 % other race/multiracial.	USDA Household Food Security Survey Module (HFSSM) ten-item survey, categories of food security – food-secure and food-insecure.	22·1 %	- FI individuals more likely to have peripheral arterial disease (PAD) than food-secure individuals.
Sattler, E. L., & Lee, J. S. (2013). Persistent food insecurity is associated with higher levels of cost-related medication nonadherence in low-income older adults. *Journal of Nutrition in Gerontology and Geriatrics*, *32*(1), 41–58. https://doi.org/10.1080/21551197.2012.722888	Georgia Advanced Performance Outcomes Measures Project – 6, 2008–2009, state, USA.	Longitudinal	The longitudinal study collected three waves of self-administered mail surveys conducted 4 months apart in 2008 and 2009 among OAANP congregate meals (CM) and home-delivered meals (HDM) participants and wait-listed individuals; subsample with at least baseline and one follow-up survey.	*n* 664, 60+ years, mean age= 74·6 years, 71·5 % female, 28·5 % males, 31 % African-American, 67·8 % White and 1·2 % other race.	Modified version of the six-item USDA HFSSM, categories of food security – persistent food-insecure, became food-insecure, persistent food-secure and became food-secure.	53·6 % at baseline.	- Persistent FI more likely to report cost-related medication non-adherence, be younger, have a lower household income, be enrolled or waitlisted in the Home-Delivered Meals programme, be in poorer health, have 4–7 chronic diseases, take seven or more prescription medications, and use more SNAP benefits than their counterparts.
Schroeder, E. B., Zeng, C., Sterrett, A. T., Kimpo, T. K., Paolino, A. R., & Steiner, J. F. (2019). The longitudinal relationship between food insecurity in older adults with diabetes and emergency department visits, hospitalisations, Hb A1c, and medication adherence. *Journal of Diabetes and its Complications*, *33*(4), 289–295. https://doi.org/10.1016/j.jdiacomp.2018.11.011	Kaiser Permanente Colorado (KPCO) members from Denver metro area (2012–2016), local, USA.	Longitudinal	‘Kaiser Permanente Colorado (KPCO) is an integrated delivery system that provides health insurance and clinical services to approximately 650 000 individuals in the metropolitan Denver area;’ subsample aged 65 years and older with diabetes who had completed at least one Medicare Total Health Assessment	*n* 2968, > 65 years, mean age = 73·5 years, 60·6 % female, 39·4 % male, 9 % Black, 23·5 % Hispanic, 61·2 % Non-Hispanic White and 4·3 % other.	Single question screening: ‘Do you always have enough money to buy the food you need?’ with a yes or no response option. Question from the Nutrition Health Screener of the Nutrition Screening Initiative, categories of food security – food-secure and food-insecure.	7·4 %	- FI individuals more likely to be female, Black or Hispanic, be current smokers, rarely or never consume alcohol, require insulin and oral medication for diabetes, be unmarried, have fair/poor health status and quality of life, and be less educated compared with food-secure individuals. - FI individuals more likely to have higher BMI, be on Medicaid, have more difficulties with performing activities of daily living, have higher HbA1c at baseline, have depression, and have urinary incontinence, problems with memory than food-secure individuals. - FI individuals more likely to utilise EDs and have hospitalisations, lower adherence to medications than food-secure individuals.
Steiner, J.F., Stenmark, S.H., Sterrett, A. T., Paolino, A.R., Stiefel, M., Gozansky, W.G., & Zeng, C. (2018). Food Insecurity in Older Adults in an Integrated Health Care System. *Journal of the American Geriatrics Society, 66(*5), 1017–1024. ·https://doi-org.ezproxy.library.unlv.edu/10·1111/jgs.15285	Kaiser Permanente Colorado (KPCO) members from Denver metro area (2012–2015), local, USA.	Retrospective cohort	Kaiser Permanente Colorado (KPCO) initiated a survey of members in Denver and other CO communities in January 2012 that included a question about food insecurity; subsample of participants 65+ years old.	*n* 50 097, 65+ years old, 82·3 % White, 7 % Hispanic, 2·6 % Black and 4 % other.	Single question screening: ‘Do you always have enough money to buy the food you need?’ with a yes or no response option. Question from the Nutrition Health Screener of the Nutrition Screening Initiative, categories of food security – food-secure and food-insecure.	5·7 %	- FI individuals more likely to be Hispanic or Black, less educated, underweight or obese, on Medicaid, women, unmarried/single, hypertensive, diabetic, and depressed than food-secure individuals. - FI individuals more likely to have poorer general, mental and physical health, poorer quality of life, generalised anxiety disorder, dental problems, physical limitations, eat fewer servings of fruits and vegetables per d, eat less than two meals a day, have unsafe living conditions, and no one to call for help than food-secure individuals.

USDA, United States Department of Agriculture; FI, food-insecure; SNAP, Supplementary Nutrition Assistance Program; ED, emergency department.

### Quality assessment

The quality of the included articles was assessed using the quality assessment tool developed by the Effective Public Health Practice Project (EPHPP). This tool is designed to evaluate the reliability, validity and biases of quantitative studies^(16)^. The quality of the included articles was separately assessed by two researchers to ensure accuracy. These two researchers then compared their results and came to agreement on any discrepancies. Studies were rated as strong, moderate or weak based on criteria established by EPHPP for components including Selection Bias, Study Design, Confounders, Blinding, Data Collection Method, and Withdrawal and Dropouts. Table 2 presents a summary of the quality assessment of included studies.

**Table 2:** Qualitative assessment of included Studies using the assessment tool Developed by the Effective Public Health Practice Project (EPHPP)

Authors (year)	Selection bias	Study design	Confounders	Blinding	Data collection method	Withdrawal and dropouts	Global rating
Afulani et al. (2015)	1	3	1	NA	1	1	Moderate
Bengle et al. (2010)	3	3	1	NA	2	2	Weak
Bhargava & Lee (2017)	1	3	1	NA	1	2	Moderate
Brewer et al. (2010)	3	3	1	NA	2	2	Weak
Books et al. (2019)	1	3	1	NA	1	1	Moderate
Brostow et al. (2019)	2	3	1	NA	1	2	Moderate
Duerr (2006)	2	3	1	NA	1	1	Moderate
Frith & Loprinzi (2018)	1	3	2	NA	1	2	Moderate
Goldberg & Mawn (2015)	1	3	1	NA	1	2	Moderate
Hernandez et al. (2017)	1	3	1	NA	1	1	Moderate
Holben et al. (2007)	2	3	2	NA	2	2	Moderate
Jackson et al. (2019)	1	3	1	NA	1	1	Moderate
Johnson et al. (2011)	1	3	1	NA	2	1	Moderate
Kihlström et al. (2019)	3	3	1	NA	1	3	Weak
Lee et al. (2011)	2	2	1	NA	1	1	Strong
Myles et al. (2016)	3	3	1	NA	1	1	Weak
Redmond et al. (2016)	1	3	1	NA	1	1	Moderate
Sattler & Lee (2013)	1	2	1	NA	1	1	Strong
Schroeder et al. (2019)	1	2	1	NA	1	1	Strong
Steiner et al. (2018)	2	2	2	NA	1	2	Strong

1, Strong; 2, Moderate; 3, Weak; NA, Not Applicable.

## Results

The search of the five electronic databases yielded 3579 potential articles. After removing exact duplicates, 3435 articles remained to be evaluated in step 1. A total of 315 articles met the inclusion criteria after their titles and abstracts were screened. The full text of these articles were screened in step 2. Of the 315 full-text articles that were screened, 295 articles were eliminated and 20 articles were retained for this review (see Fig. 1).

### Quality assessment

Of the twenty studies included in this review, twelve were rated as Moderate, four as Strong and four as Weak. Referring specifically to the twenty studies included here, the four studies classified as Strong obtained such a rating in at least one of the quality dimensions with no score lower than 2, with a score of 1 denoting Strong, a score of 2 denoting Moderate and a score of 3 denoting Weak. The twelve studies indicated as Moderate had a quality rating of 3 in at least, but not more than, one of the quality dimensions. Lastly, the remaining four studies designated as Weak had multiple quality ratings of one along multiple dimensions of quality. See Table 2 for more detailed results.

### Description of included studies (Table 1)

Fourteen of the studies included in this review were cross-sectional^(17–30)^; and three were longitudinal studies^(31–33)^. There was one retrospective study^(34)^, one exploratory study^(35)^, and a study that was both cross-sectional and longitudinal^(36)^. Most of the included studies were conducted using data from one state (*n* 12). Six studies were conducted in Georgia^(18,19,29,31,32,36)^, two in Colorado^(33,34)^, one in Florida^(28)^, one in Indiana^(35)^, one in North Carolina^(27)^ and one in Ohio^(25)^. The remaining eight studies analysed national datasets^(17,20–24,26,30)^.

Overall, 40 % of the included studies used a national dataset^(17,20–24,26,30)^, 25 % used a state dataset^(18,27,31,32,36)^ and 35 % used a local dataset^(19,25,28,29,33–35)^. Of the state datasets, four were data from the Georgia Advanced Performance Outcomes Measures Project –6 (GA Advanced POMP6)^(18,31,32,36)^, one was from the Nutrition and Function Study (NAFS)^(27)^, and one was from state data linked to the Centers for Medicare and Medicaid Services (CMS) data^(36)^. National datasets included data from the National Health and Nutrition Examination Survey (NHANES)^(20,22,23,30)^, the National Health Interview Survey (NHIS)^(17,24)^, and the Health and Retirement Study (HRS)^(21)^. Studies included adults aged 60+ years (*n* 14) or adults aged 65+ years (*n* 6); the mean age ranged from 69·8 to 78·2 years. To measure food insecurity status and/or severity, the majority of studies used one of the United States Department of Agriculture (USDA) Food Security Survey Modules (18 item = 2^(22)^, 10 item = 7^(17)^ and 6 item = 8^(18,19,21,28,29,31,32,36)^), two studies used a single screening question from the Nutrition Screening Initiative^(33,34)^, and one used a four-question survey^(27)^. Note, the four studies mentioned above using the GA Advanced POMP6 data all analyse the same sample of respondents^(18,31,32,36)^. The exception is that in one of the studies^(36)^ the authors further match the sample of respondents with the CMS data resulting in a smaller analytic sample relative to the other three studies using the GA Advanced POMP6 data (*n* 957 *v*. *n* 1594).

The sample size in the studies greatly varied, with below 500 older adults in five studies^(25,27–29,35)^, 500 to 1000 older adults in five studies^(18,19,31,32,36)^, 1500 to 2500 older adults in three studies^(22,23,30)^, 2501 to 5000 in two studies^(21,33)^, 5001 to 10 000 older adults in three studies^(20,24,26)^ and greater than 10 000 older adults in two studies^(17,34)^. In addition, a cross-sectional study design was the main limitation reported by 55 % of the studies^(17,18,20,22,23,26–30,36)^. Other reported limitations were self-reported data in 30 % of studies^(17,18,20,21,24,30)^, selection bias reported by 20 % of studies^(18,31,32,34)^ and non-generalisable results reported by 15 % of the studies^(21,29,34)^.

### Outcomes

Table 1 contains a summary of findings from all twenty studies included in this review. Food-insecure individuals were more likely to be younger^(18,19,26,31,35,36)^, less educated^(18,26,31,33,35,36)^, Black or African American^(18,19,31,34)^, female^(26,33,35)^, a current smoker^(26,33)^ and low income^(18,26)^. Food-insecure individuals were also more likely to self-report fair to poor health and have chronic conditions^(18)^ and to report three or more chronic diseases^(26)^. Moreover, individuals having co-morbidities, higher A1c, lower perceived quality of life, geriatric conditions and those taking diabetes medication were more likely to be food-insecure^(33)^. In addition, ‘… non-White race, history of a heart condition, preventative health behaviours, and especially depression and self-report of a psychiatric diagnosis were all associated with increased odds of being food-insecure’^(21)^. Being non-married, non-White, having lower educational attainment, being depressed, not having financial help and lacking insurance coverage were negatively associated with being food-secure^(23)^, and ‘marginal, low, or very low food security (was) associated with increased odds of having peripheral arterial disease …’^(30)^.

### Food insecurity and government assistance programmes

Ever receiving the Supplemental Nutrition Assistance Program (SNAP), formerly known as food stamp benefits, was associated with food insecurity in two studies^(23,31)^. Moreover, individuals on the waitlist for the Older Americans Act Nutrition Program (OAANP) were more likely to be persistently food-insecure than current participants, and participating in either meal delivery or congregate meals contributed to achieving food security^(31)^. However, results related to the impact of SNAP on food insecurity need to be viewed cautiously given the endogenous and misreported nature of SNAP participation^(37)^. In addition, individuals that were eligible for both Medicaid and Medicare were more likely to be food-insecure^(33)^, and individuals who had Medicaid insurance were more likely to be food-insecure^(34)^.

### Food insecurity and weight status

Food-insecure individuals were more likely to be obese^(26)^, have a higher BMI^(19,33)^, and waist circumference, and have arthritis, joint pain, and weight-related disability^(19)^. However, Brostow et al. (2019) found that being overweight or obese was not associated with increased odds of food insecurity^(21)^. Furthermore, Hernandez et al. (2017) found that food insecurity was not associated with weight status in women, and ‘food-insecure men had 42 % lower odds of being overweight compared with normal weight and 41 % lower odds of being overweight or obese compared with normal weight…’^(24)^.

### Food insecurity and cost-related medication use and healthcare utilisation

One study found a ‘…dose-response relationship between (food insecurity) and cost-related medication underuse (CRMU) …behaviors’ of foregoing or taking less medication and delaying refills to save money, inability to afford medication and asking a prescriber for a lower cost medication^(17)^. Another study concluded that individuals who practiced cost-related medication non-adherence were more likely to respond affirmative to questions indicating food insecurity, and ‘… food-insecure individuals were approximately 2·95 times …more likely to report (practicing cost related medication non-adherence)’^(18)^. In one study, researchers found that individuals who were persistently food-insecure and those who became food-insecure were more likely to practice medication non-adherence^(32)^. In addition, Bhargava and Lee reported that there was no significant difference in healthcare utilisation by food security status^(36)^.

### Food insecurity and mental health

Food insecurity was associated with depression in four studies^(20,21,23,27)^. Johnson et al. (2011) found that individuals who were food-insecure ‘…were almost five times as likely to report depressive symptoms compared to those who were food secure’^(27)^. Food insecurity was also associated with a self-reported psychiatric diagnosis^(21)^. One study concluded that ‘individuals who were marginally food secure, food insecure without hunger and food insecure with hunger had significantly lower cognitive function ….’^(22)^, and another study found that food insecurity was associated with cognitive restraint after controlling for confounding variables^(29)^.

### Food insecurity and physical health

Physical functioning limitations increased as food insecurity increased^(25,26)^. Moreover, all eight domain scores from a frequently used health survey measuring quality of life (SF-36) including physical functioning, physical role limitations, bodily pain, general health perceptions, energy/vitality, social functioning, emotional role limitations and mental health were associated with severity of food insecurity^(25)^. Jackson et al. (2019) found that the odds of food insecurity were greater for those with physical functioning limitations and more than three chronic diseases^(26)^. Another study found that food-insecure individuals were more likely to report ≥ 14 physically unhealthy days and ≥ 14 d with activity limitations^(28)^.

With respect to the relationships noted above, no discernable patterns emerge whereby studies classified as strong find one relationship relative to those classified as moderate or weak finding another. Most of the associations, in terms of the direction, are consistent across the studies, which individually vary in quality. The one exception is the relationship between food security and obesity/weight status where there are divergent findings across studies. However, there appears to be no pattern as it relates to the quality of study and the direction of the documented relationship. Specifically, three studies respectively classified as strong, moderate and weak all find a positive association between food insecurity and weight, whereas two studies both classified as moderate find either no relationship or an inverse relationship between food insecurity and weight. See Table 2 for a further breakdown of study quality.

## Discussion

Despite the high prevalence and the detrimental health and well-being effects of food insecurity among older adults, a limited number of studies over the past 15 years have assessed the associated factors of food insecurity in this population. Overall, this review uncovered that social determinants of health including education^(18,26,31,33,35,36)^, race and ethnicity^(18,19,31,34)^, gender^(26,33,35)^, and income^(18,26)^ were consistently associated with food insecurity. These results are consistent with previous findings of higher rates of food insecurity among lower-income older adults and those from racial or ethnic minorities^(15)^. Many of the factors associated with food security in older adults are similar in the estimated direction of the relationship to those found in other adult age groups. Lower educational attainment, lower household income, female gender, having a disability and being non-White race/ethnicity are associated factors that have been consistently documented to have a negative association with food security for decades by the USDA through the annual Current Population Survey Food Security Supplement^(38)^. Similarly, being a smoker^(39–42)^ and having poorer self-reported health^(43–46)^, chronic disease^(12)^, poor mental health outcomes^(7,11)^, and medication non-adherence^(45,47–49)^ are documented in the peer-reviewed literature to have a negative association with food security. Though limits in physical functioning are less documented in non-older adult populations, one well-established likely related factor is disability status^(38,50)^. These results suggest that upstream systemic-level interventions, though difficult to implement, may be better suited to deal with food insecurity among the senior population.

In addition, ‘younger’ older adults were found to have^(18,19,26,31,35,36)^ higher rates of food insecurity (age ranges from 60–64, 60–69, 60–74, 65–74 and 60–84 years). This is also consistent with other findings^(15,51,52)^. For example, in their report for Feeding America, Zilak & Gundersen (2020) uncovered that food insecurity rates among seniors aged 60–64 years were twice as high as seniors aged 80 years and older^(15)^. This increased likelihood of being food-insecure may be explained, in part at least, by eligibility for Medicare and other safety net programmes that help to buffer resource limitations^(51)^.

This review revealed an inconsistent relationship between food insecurity and weight status among older adults with some studies finding a link between food insecurity and obesity^(26)^, higher BMI^(19,33)^, and waist circumference^(19)^, and other studies finding no association with being overweight or obese^(21)^. This is not surprising given our understanding that human behaviour is complex, and that there exist inherent statistical issues around measurement error in both food security and obesity^(53,54)^. Specifically, the socioecological model postulates that health behaviour is influenced by factors at several levels including intrapersonal, community, organisation, government, industry and societal^(55)^. This is a model that is frequently cited in obesity research^(56)^. The relationship between food insecurity and weight status is possibly bidirectional; it is possible that food insecurity preceded obesity for some and for others it followed. Additionally, obesity is likely to occur over the long term, and people are likely to ebb and flow in and out of food insecurity^(57)^. Further, measurement error related to assessing food security and/or obesity/weight status can introduce bias in widely used parametric estimators given the non-classical nature of such misclassification. This is a reasonable concern given the vague and somewhat arbitrary nature that food security is defined and measured by the USDA, the misreporting of food security status due to perceived stigma, and/or the inexact methods to measure BMI. Given the non-classical nature of such measurement error, the estimated relationship between food insecurity and weight/obesity can be wrong in terms of magnitude as well as in the sign of the relationship. Directly confronting such measurement error becomes extremely difficult, though progress has been made in the economics literature^(58)^.

This review found that food-insecure older adults are likely to make spending trade-offs including cost-related medication non-adherence^(17,18,32)^. These results may be linked to the rise in healthcare costs. On average, Medicare enrollees spend over $5000 out of pocket annually, including over $650 on prescription drugs^(59)^. The price of prescription drugs is thought to be the driving force in the increasing cost^(60)^. ‘Since 2001, prices on prescription drugs have grown at an average annual rate of about six percent as measured by the producer price index for pharmaceuticals – a much higher rate than general inflation’^(60)^. In addition to increasing medication cost, most Medicare prescription drug plans have a coverage gap, also called the ‘donut hole’, which is a temporary limit on what the insurance plan can cover in terms of prescription drugs^(61)^. While recent reforms have shifted the structure of this gap, it still leaves many seniors potentially paying higher out-of-pocket costs, dependent on the cost of their medications and the new cost share. Given the importance of medication adherence to maintaining health, policy-level interventions aimed at drug costs to help mitigate spending trade-offs are warranted.

Food insecurity was associated with depression^(20,21,23,27)^, a self-reported psychiatric diagnosis^(21)^ and significantly lower cognitive function^(22)^. These results are consistent with recent findings. For example, Madden et al. (2020) reported that food-insecure seniors younger than 65 years of age were 2·65 times more likely to report depression, and seniors aged 65 years and older were 1·6 times more likely to report depression relative to food-secure seniors^(51)^. The relationship between food insecurity and mental health can be bidirectional, where poor health increases financial strains and food insecurity, and financial strain and food insecurity may increase the risk of poor health. Additionally, mental wellness can affect one’s ability to attain and maintain employment/steady income. This relationship is likely to be bidirectional as well, where the hardships imposed by food insecurity may result in poor mental health outcomes^(62)^. In a systematic review, Bruening et al. ‘suggest a bidirectional association whereby food insecurity increases the risk of poor emotional health, and poor emotional health increases the risk of food insecurity’^(7)^.

Most of the studies included in this review were cross-sectional in nature making it difficult to infer causality. In addition to the quality measures highlighted in Table 2 and the measurement error issues commented on earlier, readers should interpret results of the included studies cautiously given the bidirectional nature of how food insecurity and other measures of interest are determined. The consequence of estimating the effect of some independent variable (e.g. mental health) on a particular dependent variable (e.g. food insecurity) when such bidirectionality exists is the estimated effect being contaminated with simultaneity bias^(63)^. The reason for such bias stems from the failure of the assumption that the error term in regression-based models is uncorrelated with included model covariates. In addition to instrumental variables (IV) and partial identification methods using cross-sectional data, incorporating the dimension of time can potentially help in dealing with such endogeneity. With that said, if one is to incorporate lagged values as a means to avoid simultaneity, it should be done so in the context of using the lagged endogenous variable in an IV estimation strategy and only if the lagged regressor meets the criteria of being a valid exclusion restriction^(64)^. Even so, few studies have examined the relationship between food insecurity and the associated factors that were found to be significant in this review over time. This highlights the need for more longitudinal studies that would allow researchers to employ panel data methods, including causal inference methods such as difference-in-differences, to, under a specific set of assumptions, tease out the causal relationship between food insecurity and its associated factors among older adults. Additionally, 40 % of the studies included in this review used a national dataset with representative samples of older adults in the USA, thus increasing the generalisability of the results. Similarly, a majority of the studies used state or multistate datasets consisting of a representative sample of the states’ older adult population. However, 80 % of the state datasets were from Georgia. Given the wide distribution of food insecurity rates by state, studies are warranted for other states and regions that have distinct characteristics.

The sample sizes in the studies included in this review were relatively large with half of the studies including 1500 participants or more. After conducting a quality assessment, 60 % of the studies were rated as moderate quality with many studies reporting several limitations including survey tool validity and reliability, self-reported data, and selection bias. However, some studies did not include a self-evaluation of the research or a clear list of limitations. Future studies must ameliorate quality-related factors in their studies and clearly discuss limitations so that others can properly interpret and potentially replicate findings.

This review systematically assessing the associated factors of food insecurity in the USA is subject to several limitations. The inclusion criteria limited this review to studies conducted in the USA and published in English, possibly excluding relevant studies conducted elsewhere and/or published in other languages. This limits the generalisability of this review to other countries and parts of the world. Future studies may consider expanding criteria to include more countries and articles published in other languages. While this study restricted the sample to adults aged 60+ years, there are still compositional differences among the study samples; thus, attention should be paid when making comparisons. Additionally, because we limited our research to peer-reviewed articles that were published between 2005 and 2019, we may have missed relevant findings that were published in non-peer-reviewed sources or those that were published outside of our inclusion dates. And while our selection process was well defined, it is possible that others doing the screening may have resulted in the inclusion of different articles. Further, each included study is subject to its own limitations and biases. Lastly, there is no discernable pattern related to the consistency of findings by the assessed quality of the included studies.

Overall, the correlates of food insecurity among older adults identified during this review are younger age^(18,19,26,31,36)^, lower educational level^(18,26,31,33,35,36)^, Black or African American race^(18,19,31,34)^, female gender^(26,33,35)^, being a current smoker^(26,33)^, low-income^(18,26)^, fair to poor health status (self-reported), and having chronic conditions and other co-morbidities^(18,26,33)^. In addition, depression^(20,21,23,27)^, non-married status, lack of insurance coverage^(23)^, cost-related medication underuse^(17,18,32)^, lower cognitive functioning^(22)^ and physical functioning limitations^(25,26)^ were other significant correlates of food insecurity among older adults. Safety net programmes generally help to buffer some effects of food insecurity; however, individuals sometimes employ coping mechanisms that have the potential to exacerbate the issue, such as skipping or cutting medications and consuming lower nutrient foods. Future studies may want to employ a meta-analysis of such findings to provide a more precise estimate of the effects of food insecurity on the health and well-being of seniors. Public health interventions should be upstream and systemic to address the underlying determinants of food insecurity.
